# Role of the PADI family in inflammatory autoimmune diseases and cancers: A systematic review

**DOI:** 10.3389/fimmu.2023.1115794

**Published:** 2023-03-20

**Authors:** Changhui Zhu, Chunyan Liu, Zhengbin Chai

**Affiliations:** ^1^ Department of Plastic Surgery, Shandong Provincial Qianfoshan Hospital, School of Basic Medical Sciences, Weifang Medical University, Weifang, Shandong, China; ^2^ Shandong Provincial Key Laboratory for Rheumatic Disease and Translational Medicine, The First Affiliated Hospital of Shandong First Medical University & Shandong Provincial Qianfoshan Hospital, Jinan, China; ^3^ Department of Clinical Laboratory Medicine, Shandong Public Health Clinical Center, Shandong University, Jinan, China

**Keywords:** PADI, citrullination, autoimmune, cancer, inflammatory

## Abstract

The peptidyl arginine deiminase (PADI) family is a calcium ion-dependent group of isozymes with sequence similarity that catalyze the citrullination of proteins. Histones can serve as the target substrate of PADI family isozymes, and therefore, the PADI family is involved in NETosis and the secretion of inflammatory cytokines. Thus, the PADI family is associated with the development of inflammatory autoimmune diseases and cancer, reproductive development, and other related diseases. In this review, we systematically discuss the role of the PADI family in the pathogenesis of various diseases based on studies from the past decade to provide a reference for future research.

## Highlights

1. SNP of PADI family can endow disease susceptibility.
2. The citrullination of PADI family makes it participate in different physiological processes.
3. The role of PADI family in various physiological processes makes it participate in the occurrence of human diseases.
4. PADI family can be used as the target of disease treatment for drug development.

## Introduction

The peptidyl arginine deiminase (PADI) family is composed of isozymes with sequence similarity and is located on human chromosome 1p36.13. Its family members include PADI1-4 and PADI6. The PADI family is a class of calcium ion-dependent enzymes that can catalyze the citrullination of proteins. It converts positively charged arginine into neutrally charged citrulline, thereby changing the structure and function of proteins. Studies have shown that PADI family members have unique subcellular localization and tissue distribution, which determine the functional specificity of each family member (**Table 1**).

**Table 1:** Distribution and function of PADI family members.

PADI family	Subcellular localization	Tissue distribution	The transcription factor that regulates PADI	Mechanism	Function
PADI1	cytoplasm	epidermis uterus	NF-κB (1) MZF1 Sp1/Sp3 (2)	citrullinated intermediate filaments, keratin and filamentous proteins	Promote the differentiation of keratinocytes (3–6)
				chromatin structure is regulated by citrullinated histones	To ensure the smooth development of early embryos (7)
PADI2	cytoplasm nucleus mitochondria (8, 9)	brain skeletal muscle secretory glands inflammatory cells	Sp1/Sp3 (10) P2X7 (11) SOX9 FOXL2 (12)	catalyzes the citrullination of histone and nonhistone proteins	Forming NETs (13–16) Citrullination of myelin basic protein in brain tissue (17)
PADI3	cytoplasm	keratinocyte follicles	NF-Y Sp1/sp3 (18)	citrullinated cytokeratin K1, K 10 and filaggrin	Promote epidermal homeostasis and barrier formation (3)
PADI4	cytoplasm nucleus	granulocytes, cancer cells	NF-κB (19) P53 (20) activator protein-1 Sp1 nuclear factor-Y (21)	chromatin structure is regulated by citrullinated histones	Forming NETs (22, 23)
PADI6	cytoplasm	embryo oocyte	Nobox (24) sp1 (25)	Promote the formation of cytoplasmic lattice in oocytes	Ensure the normal development of the early embryo (26)

As shown in **Table 1**, the specific distribution of the PADI family in tissues determines that the PADI family can participate in different physiological processes, which was further validated in single-cell studies of the PADI family. Downregulation of PADI1 and PADI3 in the skin of the extremities leads to alterations in filaggrin and keratin. PADI2 is associated with multiple sclerosis (MS) and posttreatment Lyme disease. PADI4 can induce arteriosclerosis by mediating the formation of NETs or promote tumor growth and metastasis by altering the tumor microenvironment. Furthermore, PADI6 is associated with the ovarian reserve (OR), oocyte maturation and early embryonic development (27–32).

SNP analysis of the PADI family showed that genetic variations in the PADI gene were significantly associated with susceptibility to multiple diseases. This allows the PADI family to participate in the development of multiple diseases, as shown in **Table 2**.

**Table 2:** SNP analysis of the PADI family.

PADI family	SNP sites	Disease susceptibility
PADI2	rs1005753, rs2057094, rs2076616, rs2235912, rs2235926	rheumatoid arthritis (33–36)
PADI4	rs11203367, rs2240335, rs2240340, rs1748033, rs874881 rs11203366, rs2240339, SCV000804840, SCV000807675 rs2240335, PADI4 _ 94, PADI _ 104, PADI4 _ 92 ADI4-94G/A, PADI4-92C/G, PADI4-90C/T PADI4 _ 89 × G、 PADI4 _ 90 × T 和 PADI4 _ 92 × G	rheumatoid arthritis (37–48)
	rs1635564, rs2240340, rs1929992	systemic lupus erythematosus (49, 50)
	rs11203366, rs11203367, rs874881, rs2240340, rs11203368	systemic lupus erythematosus lupus nephritis (49)
	rs10437048, rs41265997, rs2501796, rs2477134	esophageal cancer (51)
	rs874881, rs11203366, rs11203367, rs2240339 SCV000804840, SCV000807675	osteoarthritis (40)
	rs1748033	autoimmune thyroid diseases (52)
	rs1635566, rs882537	gastric carcinoma (53)
	rs2240337G > A, rs11203366, rs1886302, rs1635562, rs1635564, rs2477137	esophageal squamous cell carcinoma (54)

Therefore, PADI family members may be associated with the occurrence of diseases by participating in different physiological processes. The current research shows that the PADI family mainly participates in the occurrence of inflammatory autoimmune diseases, cancer and reproductive development-related diseases by participating in gene expression regulation, NETosis, the secretion of inflammatory cytokines, energy metabolism and the release of extracellular vesicles. The pathological mechanisms by which the PADI family acts in various diseases has never been systematically described before. In this paper, we reviewed the progress of research on the PADI family in the past decade to provide a reference for further study on the role of the PADI family in diseases, thereby promoting the application of the PADI family in the clinical treatment of human diseases.

## Physiological processes involving the PADI family

### Gene expression regulation

First, the PADI family can regulate chromatin status through the citrullination of histones to activate transcription. PADI2 can prevent the degradation of androgen receptor (AR), mediate H3R26_Cit_, promote the binding AR and its target gene, and promote the transcriptional activation of the target gene (55).

Second, the PADI family mainly regulates the state of chromatin by crosstalk between the citrullination of histones and the methylation of histones, thus controlling gene transcription. During the activation of estrogen receptor (ER) target genes, ER recruits PADI2 to the promoter of the target gene, and PADI2 catalyzes H3R26Cit to agglutinate chromatin. Thereafter, H3K27 demethylase is recruited to chromatin, leading to the transcriptional activation of ER target genes (56–58). It has also been shown that H3R26_Cit_ mediated by PADI4 interacts with H3K27me3 (59). When PADI4 regulates the expression of p53 target genes, the interaction between PADI4 and p53 leads to the recruitment of PADI4 to the p21 promoter. This increases histone citrullination and reduces histone Arg methylation, thereby inhibiting the expression of p21, cell cycle arrest and apoptosis (60). It has also been shown that PADI2/3 can inhibit the premature differentiation of mouse trophoblastic stem cells by maintaining key DNA methylation sites (61).

In addition, the PADI family can also regulate gene transcription by acting on enzymes involved in the transcription process. The PADI2-mediated citrullination of RNA polymerase II C-terminal domain (RNAP2-CTD) R1810 facilitates RNAP2 pause release and the efficient transcription of RNAP2 (62). However, the citrullination of DNA methyltransferase DNMT3A by PADI4 increases the level of DNA methyltransferase 3A (DNMT3A). This leads to the hypermethylation of certain gene promoters, thus affecting transcription (63).

### Neutrophil extracellular trap formation

**Figure 1:**
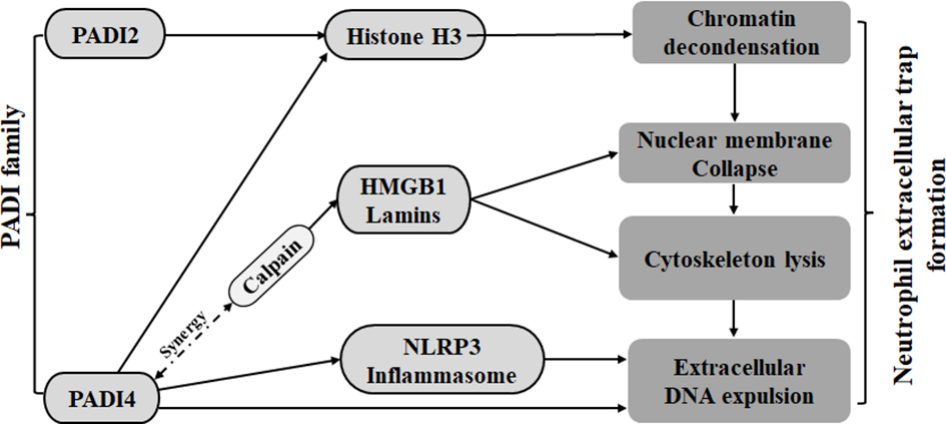
The PADI family promotes the formation of neutrophil traps. Both PADI2 and PADI4 can cause chromatin depolymerization by acting on histone H3. PADI4 can cooperate with calpain on HMGB1 and lamins to cause nuclear membrane disruption. PADI4 can also mediate nuclear membrane disruption and cytoskeleton fragmentation by promoting nucleosome assembly.

NETosis is a type of reticular DNA structure that contains histones and cytotoxic proteins that are formed by neutrophils through the penetration of the plasma membrane, the decomposition of the cytoskeleton and nuclear membrane, the concentration of chromatin, and the assembly of antibacterial proteins on chromatin scaffolds when the body is stimulated by foreign invaders. Research shows that PADI4 can participate in all aspects of neutrophil extracellular traps (NETs) (64). First, PADI2 and PADI4 can cause chromatin deagglutination through citrullinated histone H3 (CitH3) and promote the release of DNA out of cells (13–16, 22, 23). Second, PADI4 can also mediate the degradation of laminin and HMGB1 through a synergistic effect with calpain, leading to nuclear membrane rupture (65). In addition, PADI4 can also participate in the decomposition of nuclear and plasma membranes by promoting the assembly of NLRP3 inflammatory bodies (66). Therefore, PADI2 and PADI4 in the PADI family can promote NETosis (**Figure 1**).

### Secretion of inflammatory cytokines

First, the PADI family can affect cytokine secretion by participating in the differentiation and apoptosis of immune cells. In activated Jurkat cells, overexpression of PADI2 citrullinates the surface vimentin, thereby inducing the apoptosis of activated Jurkat cells (67). During the differentiation of Th cells, PADI2 can inhibit the differentiation of Th2 cells through citrullinated GATA3, thereby inhibiting the secretion of interleukin 4 (IL-4), IL-5 and IL-13. PADI2 can also promote the differentiation of Th17 cell ROR through the citrullination of RORγt, thereby promoting the secretion of IL-17A and IL-17F (68). In macrophages, PADI2 can promote the expression of IL-1β, IL-6 and TNF-α through the citrullination of NF-κB p65 (69). PADI4 can positively regulate TNF-α and CCL2 (70). PADI2 coordinates with PADI4 to regulate the assembly of the NLRP3 inflammasome to promote IL-1β release (71). In the process of macrophage differentiation, PADI2 and PADI4 lead to citrullinated PAI-2, separating PAI-2 from PSMB1. This leads to the upregulation of PAI-2 to promote the secretion of TNFα and IL-1β (72).

**Figure 2:**
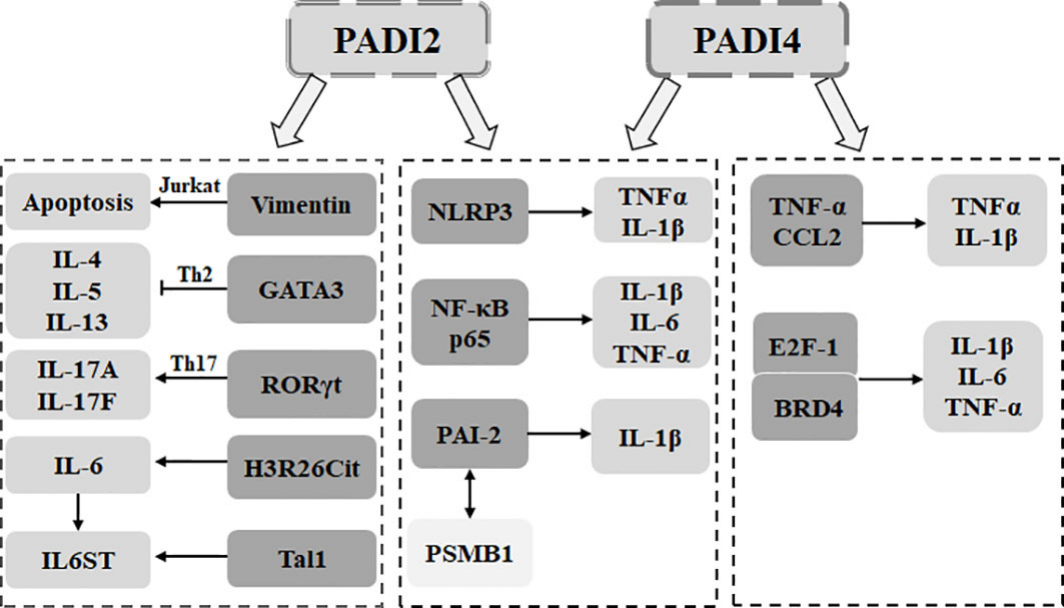
The PADI family is involved in the secretion of inflammatory cytokines. In immune cells, PADI2 can mediate Jurkat apoptosis through the citrullination of vimentin. PADI2 can be used to citrullinate GATA3. RORγt determines the differentiation direction of Th cells and affects the secretion of inflammatory cytokines. PADI4 can directly affect the secretion of inflammatory cytokines in macrophages. PADI2 can cooperate with PADI4 to affect the assembly of macrophage inflammatory bodies, mediate the dissociation of PAI-2 and PSMB1, and promote the secretion of inflammatory cytokines. In nonimmune cells, PADI2 can promote the secretion of inflammatory cytokines by targeting histones and can also act as an epigenetic coactivator to promote the signal transduction of inflammatory cytokines. PADI4 can promote the binding of BRD4 and E2F-1 and promote the secretion of inflammatory cytokines. PADI4 can also target NF-κB p65 to promote the secretion of inflammatory cytokines.

Second, the PADI family regulates the secretion of inflammatory cytokines by affecting nonimmune cells. In bone marrow mesenchymal stem cells, PADI2 can increase the level of IL-6 by mediating H3R26Cit (73). PADI4 can be used as an epigenetic coactivator of Tal1 to activate the expression of IL6ST, a target gene of Tal1/PADI4. This promotes cytokine signal transduction, including that of IL-6 (74). PADI4 can promote the binding of bromodomain containing protein 4 (BRD4) and the cytokine gene promoter E2F-1 through citrullination and promote the secretion of TNFα, CCL3 and IL-1β (75). PADI4 can also promote the expression of IL-1β and TNFα through the citrullination of NF-κB p65 (76). Therefore, PADI2 and PADI4 of the PADI family can promote the secretion of inflammatory cytokines (**Figure 2**).

### Energy metabolism and extracellular vesicle release

Studies have shown that the PADI family can participate in energy metabolism. PADI1 and PADI3 can promote glycolysis through citrulline pyruvate kinase M2 (PKM2) arginine 106, leading to the proliferation of cancer cells (77, 78). In addition, TINCR promotes *de novo* lipid biosynthesis and histone H3K27 acetylation by preventing the ubiquitination and degradation of ACLY, leading to the accumulation of acetyl-CoA in cells. Additionally, it mediates the drug resistance of tumors through the PADI-MAPK-MMP2/9 pathway (79). Therefore, the PADI family can participate in intracellular energy metabolism.

Extracellular vesicles (EVs) are a heterogeneous group of vesicles; they contain various proteins, lipids and nucleic acids. Studies have shown that PADI2, PADI3 and PADI4 in the PADI family promote the carcinogenic microenvironment by mediating the formation of EVs, leading to tumor invasion (80, 81). Therefore, the PADI family can participate in the formation of extracellular vesicle release.

Recent research has shown that the PADI family can participate in the occurrence of diseases by participating in various physiological processes. Next, we will discuss the molecular mechanisms by which the PADI family acts in various diseases.

## The PADI family and inflammatory autoimmune diseases

**Figure 3:**
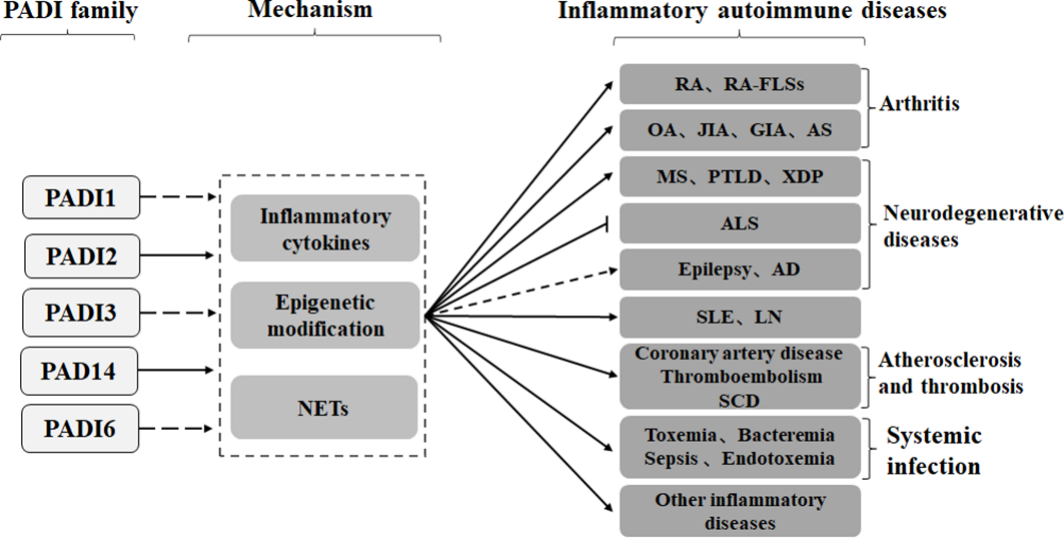
PADI2 and PADI4 in the PADI family can promote inflammatory autoimmune diseases, such as arthritis, neurodegeneration, arteriosclerosis and thrombosis, systemic lupus erythematosus and infection, through cytokines, epigenetics and NETs. No studies have shown that other members of the PADI family, other than PADI2 and PADI4, are involved in the development of inflammatory autoimmune diseases. Recent studies have shown that the PADI family can inhibit the development of ALS, but the role of the PADI family in epilepsy and AD is still controversial.

Studies have shown that the PADI family can mediate the formation of autoantibodies through epigenetic modifications, protein posttranslational modifications, NETosis and cytokine production, thus participating in the occurrence of inflammatory autoimmune diseases such as arthritis, neurodegenerative diseases, atherosclerosis and thrombosis, systemic lupus erythematosus, and infection (**Figure 3**).

### Arthritis

Arthritis is a general term that can be applied to numerous conditions. It is an inflammatory disease that occurs in the joints and surrounding tissues of the human body. Studies have shown that the PADI family is an important participant in the occurrence of arthritis (82).

The PADI family promotes the occurrence and exacerbation of rheumatoid arthritis (RA) by acting on fibroblast-like synovial cells (FLSs). Moreover, PADI4-mediated NETosis results in the release of high levels of PADI into the spinal fluid (SF) of RA patients (83). However, hypoxic conditions in RA can further upregulate the levels of PADI2 and PADI4, leading to an increase in citrullinated fibrinogen in FLSs (84, 85). Since PADI4 can provide more citrullinated epitopes than PADI2, high titers of anti-citrullinated protein antibodies (ACPAs) preferentially bind to citrullinated fibrinogen catalyzed by PADI4, which also leads to the preferential binding of ACPAs to histone H3 mediated by PADI4 (86, 87).

The binding of PADI4 to histone H3 in the p21 promoter region leads to the inhibition of p21 transcription and apoptosis in RA-FLSs. This promotes hypoxia-induced autophagy and proliferation and exacerbates the malignant progression of RA (85, 88). PADI4 can also increase H3 citrullination in CD14^hi^ monocytes through TLR and induce the expression of TNFα, MIP1β, IFNα, and IL-12 and the formation of monocyte extracellular traps (METs). Therefore, inflammation and the production of ACPAs are promoted and RA is exacerbated (89). The citrullination of fibrin mediated by PADI2 can induce the expression of the proinflammatory cytokines IL-6 and IL-8 through the TLR4 pathway. This leads to an inflammatory response in RA synovial fibroblasts (RASFs) and promotes the occurrence of RA (90). Furthermore, the dysregulation of PTPN22 can also lead to the aggravation of RA, because PTPN22 can inhibit the high citrullination mediated by PADI2 and PADI4 (91, 92).

In addition, the PADI family can exacerbate RA through other mechanisms. In synoviocytes, the interaction of PADI4 with SYVN1 may induce cell proliferation by inhibiting the p53 pathway and apoptosis and may also trigger RA by mimicking the state of endoplasmic reticulum stress inhibition (93). Citrullination of fibronectin (FN) by PADI4 leads to the upregulation of the proteolytic activity of ADAMTS4, which promotes the erosion of ADAMTS4 in joints. This causes the destruction of cartilage and the aggravation of RA (94). Furthermore, individual genetic polymorphisms of PADI2 and PADI4 can increase susceptibility to RA (35–37, 41, 43, 46, 47, 95–97). In ACPA RA, PADI4 can help HLA-DRB1 bind to ACPA (98, 99). Studies have shown that the upregulation of PADI2 and citrullination of the protein are associated with RA-related interstitial lung disease (RA-ILD), but the specific mechanism still needs further study (100, 101).

The PADI family also plays an important role in other types of arthritis. PADI4 polymorphisms confer susceptibility to OA and juvenile idiopathic arthritis (JIA) (40, 102, 103). Additionally, PADI4 can promote the progression of glucose 6-phosphate isomerase-induced arthritis (GIA) by mediating NETosis, increasing the number of CD4+ T and Th17 cells, and upregulating the level of IL-6 (104). In ankylosing spondylitis (AS), the upregulation of PADI4 mediates TNF-α-induced proliferation and the osteogenic differentiation of human mesenchymal stem cells (hMSCs), aggravating the progression of AS (105).

In summary, the current study shows that PADI2 and PADI4 of the PADI family play a role in promoting arthritis, but whether other members of the PADI family are associated with arthritis remains to be elucidated.

### Neurodegenerative diseases

Neurodegenerative disease is a general term for a class of diseases in which a large loss of neurons and/or their myelin sheaths leads to neural dysfunction, including epilepsy, Alzheimer’s disease (AD), Parkinson’s disease (PD), and amyotrophic lateral sclerosis (ALS). Studies have shown that the PADI family can influence neurodegenerative disease progression through epigenetic modifications and autoantibodies.

On the one hand, the PADI family promotes the occurrence of neurodegenerative diseases. In the hippocampus of AD patients, upregulation of PADI2 leads to the abnormal accumulation of citrullinated GFAP, promoting AD progression (106). Moreover, high PADI2 expression in the CNS leads to the exacerbation of MS and posttreatment Lyme disease (PTLD) (28). In prion-infected astrocytes, the upregulation of PADI2 activates the overexpression of citrullinated proteins. This leads to a functional change in enolase and promotes the malignant progression of prions (107). Moreover, X-linked dystonia Parkinson’s disease (XDP) is aggravated by increased levels of PADI2, PADI4, CitH3 and inflammation in the prefrontal cortex (PFC) and its derived fibroblasts (108).

On the other hand, the PADI family inhibits the development of neurodegenerative diseases. First, PADI family members can prevent disease by maintaining nerve cell homeostasis. The citrullination of proteins by PADI2 promotes chromatin decondensation. This leads to the upregulation of oligodendrocyte differentiation genes, ensures normal oligodendrocyte differentiation, myelination, and motor function, and prevents motor dysfunction (109). Moreover, in human neural stem cells (hNSCs), PADI3 binds to apoptosis-inducing factor (AIF) and translocates it to the nucleus to induce apoptosis (110). Second, the aberrant expression of the PADI family under pathological conditions prevents disease progression. In AD, PADI4 mediates autophagy and inhibits phosphorylation of the Akt/mTOR pathway, thereby increasing cell viability, inhibiting apoptosis and senescence, and delaying AD progression (111). In ALS, PADI4 citrullinates the RGG motif of the FET protein, inhibiting the aggregation of the FET protein and reducing susceptibility to ALS (112).

In addition, some studies have noted that the PADI family can participate in the occurrence of epilepsy through epigenetics and autoantibodies, but its specific mechanism of action requires further study (113).

Overall, the PADI family can both promote and inhibit the occurrence of neurodegenerative diseases. However, there are few studies on the PADI family and neurodegeneration, and further research is needed to elucidate the mechanisms of the PADI family in specific neurodegenerative diseases.

### Atherosclerosis and thrombosis

Atherosclerosis is a condition in which plaque-like deposits of lipids (atheromas or atherosclerotic plaques) form in the arterial walls of medium or large arteries, reducing or blocking blood flow. Studies have shown that the accumulation of PADI4-mediated NETosis at the site of intimal injury destroys the integrity of the vascular intima and promotes the occurrence of atherosclerosis, which is associated with coronary artery disease (CAD) (114, 115).

Intimal damage from atherosclerosis leads to thrombus formation, which is promoted by NETosis that is mediated by the upregulation of PADI4. In carotid arteries, PADI4 can promote plaque instability through NETs (116). During vaso-occlusive crisis (VOC), the upregulation of PADI4 promotes immune thrombosis through NETosis, leading to venous thromboembolism and sickle cell disease (SCD) (117). In heparin-induced thrombocytopenia (HIT) mice, PADI4 enhances neutrophil-endothelial cell adhesion and neutrophil clot infiltration through NETosis, thereby promoting the formation and progression of venous thrombosis (118). In placentation, PADI4 can promote an inflammatory response and thrombosis by mediating NETosis, increasing the susceptibility to miscarriage (119). PADI4 can also exacerbate anti-neutrophil cytoplasmic antibody-associated vasculitis (ANCA-AAV) (120).

Taken together, these results indicate that the PADI family can promote atherosclerosis and thrombosis, leading to the occurrence of related diseases.

### Systemic lupus erythematosus

Systemic lupus erythematosus (SLE) is an autoimmune disease characterized by many autoantibodies in serum and involves multiple organs. Studies have shown that the PADI family may be involved in SLE through different mechanisms.

In TLR7-induced lupus, PADI2 and PADI4 promote the induction of TLR7 in lupus through innate and adaptive immunity (121). Moreover, the upregulation of PADI4 caused by the stimulation of TLR7 promoted the phosphorylation of p38 MAPK and upregulated the expression of the P38 MAPK scaffold protein JLP. Thus, the renal invasion of neutrophils was promoted, and lupus was exacerbated (122). Mutations in the deubiquitinase domain of TNFAIP3 (A20) led to the upregulation of PADI4. This promoted protein citrullination and NETosis formation, leading to increased susceptibility to SLE (123). PADI4 polymorphisms also confer susceptibility to SLE and lupus nephritis (LN) (49).

### Systemic infection

Systemic infection is caused by a weakened immune function of the body, which leads to the infection of pathogenic bacteria and their toxic metabolites through lymphatic vessels or directly into the bloodstream. Systemic infection can be divided into toxemia, bacteraemia, sepsis, and endotoxemia. Studies have shown that the expression of the PADI family, the concentration of H3, and NETosis are positively correlated with the severity of sepsis, septic shock, and toxemia. This observation is a result of the upregulation of PADI2 and PADI4 when the body is infected. Additionally, CitH3 can promote NETosis and increase the inflammatory response, thereby promoting the aggravation of various infections (13, 14, 16, 124–127). Moreover, in sepsis, PADI2 can promote Caspase-11-dependent pyroptosis and reduce the antibacterial activity of macrophages, thereby exacerbating sepsis (15). In conclusion, the current study shows that the PADI family acts as a facilitator of systemic infection.

### Other inflammatory autoimmune diseases

First, the PADI family can promote the occurrence of other inflammatory autoimmune diseases by mediating NETosis. After renal ischemia-reperfusion (IR) injury, PADI4 is upregulated in proximal renal tubules. Moreover, PADI4 can promote tubular NF-κB activity and inflammation by citrullinating nuclear factor kappa B essential modulator (NEMO); it can also aggravate tubular inflammation and injury after IR by increasing neutrophil infiltration, neutrophil trap formation, apoptosis and the secretion of inflammatory factors (128–131). In hidradenitis suppurativa (HS), increased NETs mediated by the PADI family promote immune dysregulation and lead to inflammation (132). Respiratory syncytial virus (RSV) induces NETosis through histone citrullination by PADI1-4, leading to the development of RSV bronchiolitis inflammation in infants and young children (133). When chronic eyelid inflammation (blepharitis) occurs in the eye, PADI4-mediated aggregated NETs block the meibomian gland (MG), leading to meibomian gland dysfunction (MGD) (134). In diabetes, the upregulation of PADI4 in neutrophils increases CitH3 levels, promotes NETosis, and inhibits wound healing (135). PADI4 also promotes gallstone formation through NETosis (136).

Second, the PADI family can also promote the occurrence of inflammatory autoimmune diseases through other mechanisms. In periodontitis, increased expression of PADI2, PADI4, and citrullinated proteins accompanies the exacerbation of periodontitis (137–139). In hepatic stellate cells (HSCs), the PADI2-mediated abnormal accumulation of citrullinated GFAP promotes liver fibrosis (140). During human rhinovirus (HRV) infection, the upregulation of PADI2 in human bronchial epithelial cells leads to the citrullination of human cathelicidin LL-37. This reduces its antiviral activity against HRV and allows HRV to escape the immune response (141). Moreover, citrullinated fibrinogen (cFBG) has been detected in patients with inflammatory diseases. cFBG produced by PADI2 not only inhibits fibrin polymerization but also damages fibrin fibre properties, leading to adverse effects on hemostasis (142).

## The PADI family and cancer

**Figure 4:**
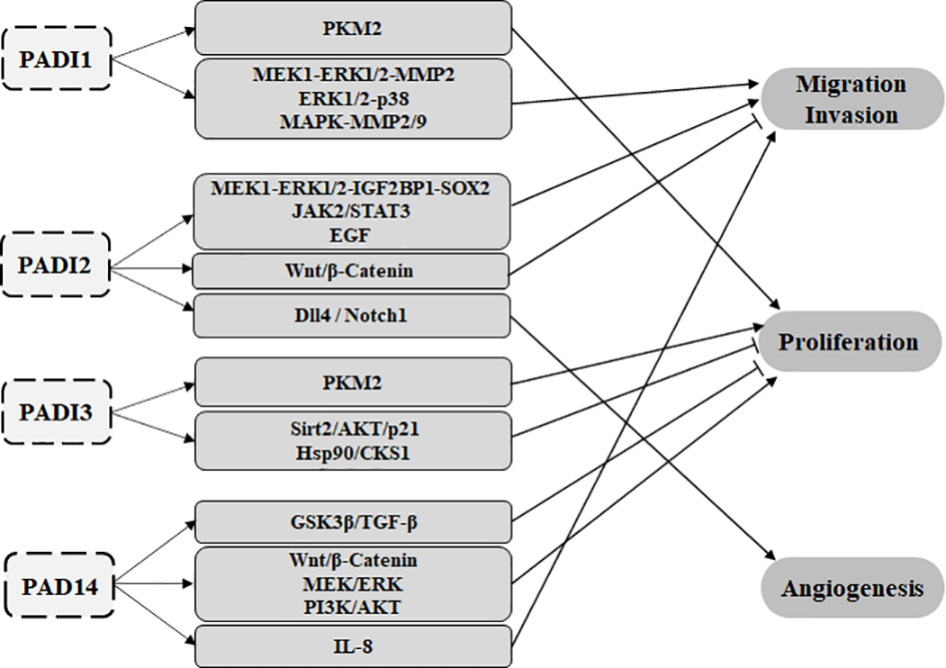
The PADI family can participate in tumour proliferation, migration, invasion and angiogenesis through various signalling pathways. PADI1 and PADI3 can promote glycolysis through the citrullination of PKM2, leading to the proliferation of tumour cells. PADI1 can promote tumour migration and invasion through MEK1-ERK1/2-MMP2, ERK1/2-p38 and MAPK-MMP2/9. PADI2 can promote tumour migration through the MEK1-ERK1/2-IGF2BP1-SOX2, JAK2/STAT3 and EGF signalling pathways. PADI2 also affects Wnt/β-catenin to inhibit tumour metastasis. PADI2 can promote tumour angiogenesis through the Dll4/Notch1 signalling pathway. PADI3 can inhibit tumour proliferation through Sirt2/AKT/p21 and Hsp90/CKS1. PADI4 can act on IL-8 to promote tumour cell metastasis. PADI2 can also affect Wnt/β-Catenin, MEK/ERK and PI3K/AKT to promote the proliferation of tumour cells. PADI4 can also inhibit the proliferation of tumour cells through GSK3β/TGF-β. At present, there is no report on PADI6 and tumours.

Studies have shown that in different cancer types, the PADI family can participate in tumorigenesis as an epigenetic modifier. It can also play a role in tumor cell proliferation, migration, invasion, angiogenesis, and drug resistance by regulating different signaling pathways, and as a result, it can act as an oncogene or tumor suppressor gene in cancer (**Figure 4**). At present, PADI2 and PADI4 are the most frequently studied members of the PADI family in tumors. In this article, we elaborate on the molecular mechanisms by which each PADI family member participates in tumors.

### PADI1

First, PADI1 can affect the proliferation of tumor cells by participating in energy metabolism. The citrullination of Arg 10 in PKM2 by PADI1 can lead to an increase in glycolysis, thus promoting the proliferation of cancer cells (77, 78). Second, PADI1 can promote EMT and metastasis of triple-negative breast cancer cells by regulating MEK1-ERK1/2-MMP2 signal transduction (143). In pancreatic ductal adenocarcinoma (PAAD), PADI1 can activate ERK1/2-p38 signal transduction, thereby promoting cell migration and invasion (140). PADI1 can also mediate the proliferation, metastasis and cisplatin resistance of nasopharyngeal carcinoma (NPC) through the TINCR-ALY-PADI1-MAPK-MMP2/9 axis (144). However, although some studies have shown that PADI1 is a poor prognostic factor of artificial cancer in pancreatic cancer and clear renal cell carcinoma and that PADI1 can also participate in the progression of laryngeal squamous cell carcinoma (LSCC), its specific mechanism of action still needs further study (145–147). Therefore, PADI1 mainly acts as an oncogene.

### PADI2

First, PADI2 can affect the proliferation of tumor cells by regulating gene expression. When normal breast cells are transformed into malignant tumors, the upregulated expression of PADI2 affects the expression of cell cycle genes, such as p21, GADD45α and Ki67, which are related to tumor progression (148). This may be because PADI2 can affect gene expression through epigenetic modifications in breast cancer. For example, in estrogen receptor ER+ breast cancer, PADI2 can not only promote susceptibility but can also citrullinate histone H3R26, change the structure of nucleosomes, promote the binding of ER and DNA, lead to the activation of ER target genes, and increase the survival rate of ER+ breast cancer patients (57). PADI2 can also citrullinate R1810 (cit1810) at RNAP2-CTD (RNA polymerase II), promote the interaction with the P-TEFb (positive transcription elongation factor b) complex, lead to the release of RNAP2, and promote the transcription of cell cycle genes and the proliferation of breast cancer cells (62). In prolactinomas and growing prolactinomas, PADI2 and PADI4 can promote the upregulation of the HMGA1, N-MYC and IGF-1 oncogenes by catalyzing the citrullination of histones, thus promoting the proliferation of cancer cells (149). In hepatocellular carcinoma, the downregulation of PADI2 inhibits EPO expression and promotes the proliferation and migration of hepatocellular carcinoma cells (150). PADI2 can also inhibit the proliferation of colon cancer cells by inducing G1 phase arrest in colon cancer cells through its citrullination effect (151).

Second, PADI2 can mediate the migration and invasion of tumor cells. PADI2 can participate in tumor cell migration and invasion by regulating gene expression. The overexpression of PADI2 can upregulate the expression of ACSL4 and BIRC3, downregulate the expression of CA9, promote abnormal lipid metabolism and tumor cell invasion, and lead to the abnormal migration of breast tumor cells (152). The downregulation of PADI2 can lead to the low expression of CXCR2, thus inhibiting the proliferation and migration of gastric cancer cells and promoting apoptosis (150). In bone marrow mesenchymal stem cells (BMMSCs) from multiple myeloma (MM), the overexpression of PADI2 promotes the expression of interleukin-6 (IL-6) through the citrullination of histone H3R26. It also mediates the drug resistance of MM and leads to the malignant progression of MM (73, 153). In skin cancer, the overexpression of PADI2 can lead to the malignant progression of tumors by promoting the inflammatory microenvironment (154, 155).

PADI2 can also participate in the migration and invasion of tumor cells by mediating the transmission of some signaling pathways. In endometrial carcinoma, PADI2 can citrullinate MEK1 arginine 113/189, promote the phosphorylation of extracellular signal regulated protein kinase 1/2 (ERK1/2), activate insulin-like growth factor II binding protein 1 (IGF2BP1), and prevent the degradation of SOX2 mRNA. This causes the abnormal accumulation of SOX2 and leads to the malignant progression of EC (156). In ovarian cancer, PADI2 can lead to metastasis and invasion by promoting the JAK2/STAT3 signaling pathway (157). In colorectal cancer (CRC), PADI2 can citrullinate β-catenin, leading to its degradation. Thus, Wnt signaling is inactivated, and the progression of CRC is inhibited (158). In breast cancer, PADI2 can mediate cell migration by promoting the EGF signaling pathway (159). However, studies have shown that the overexpression of PADI2 can promote liver metastasis of CRC (160).

PADI2 is also involved in tumor cell migration and invasion by mediating EVs. High expression of PADI2 and PADI3 can reduce the expression of moesin, increase extracellular vesicles (EVs) to release tumor-promoting factors, reduce EV tumor suppressors, and increase the invasiveness of tumor cells, thereby promoting the malignant progression of PDAC (80). The specific mechanisms of action of PADI2 in the metastasis and invasion of bladder cancer still needs further study (161).

In addition, PADI2 can mediate tumor angiogenesis. In malignant glioma, hypoxia can induce the upregulation of the PADI family (PADI1, 2, 3 and 4), and PADI2 can citrullinate vascular endothelial growth factor receptor 2 (162). Studies have shown that PADI2 is an angiogenesis-regulating gene that can promote angiogenesis through Dll4/Notch1 signaling (163, 164).

It has been reported that the overexpression of PADI2 can increase tamoxifen resistance in breast cancer cells (165).

As mentioned above, the role of PADI2 in tumors is still controversial. However, because PADI2 can participate in tumor progression by mediating tumor proliferation, migration and invasion, tumor angiogenesis and drug resistance, it has become an important target in tumor treatment.

### PADI3

As previously mentioned, PADI3 can promote glycolysis through the citrullination of PKM2, leading to the proliferation of cancer cells. PADI3 can inhibit the development of colon cancer by inhibiting the expression of Sirt2 and upregulating p21. These effects lead to a reduction in AKT phosphorylation and the downregulation of Snail, thereby inducing cell cycle arrest and inhibiting cell proliferation (166). PADI3 can also exert its antitumor activity by inhibiting the expression of Hsp90 and CKS1 (167). PADI3 can also increase the invasiveness of tumor cells by mediating EVs, thereby promoting the malignant progression of PDAC (80). Therefore, PADI3 could play a role as both an oncogene and tumor suppressor gene in tumors.

### PADI4

First, PADI4 can mediate the occurrence of cancer. PADI4 can downregulate the expression of NANOG and OCT4, the two main transcription factors of stem cells, by reducing H3R17me2a, leading to a reduction in breast cancer stem cell activity (168). PADI4-mediated NETs can help pancreatic cancer cells cross cell cycle checkpoints and promote the occurrence of PDAC (169).

Second, PADI4 can mediate the proliferation and metastasis of cancer cells. PADI4 can participate in the proliferation and metastasis of cancer cells by regulating gene expression. In oral squamous cell carcinoma (OSCC), PADI4 can upregulate its target HIST1H1B through epigenetic modifications, promoting the loss of cell differentiation and leading to the progression of OSCC (170). In esophageal squamous cell carcinoma (ESCC), PADI4 can stimulate the growth of ESCC cells and upregulate CA9 to promote ESCC metastasis (171). PADI4 synergizes with B-cell-specific Moloney leukemia virus insertion site 1 (Bmi-1) to promote the carcinogenesis and progression of ESCC (172). In prolactinomas and growing prolactinomas, PADI2 and PADI4 can promote the proliferation of cancer cells by catalyzing the citrullination of histones and upregulating the oncogenes HMGA1, N-MYC and IGF-1 (149). In lung cancer, the upregulation of PADI4 can inhibit the expression of IRF5 and CD86 and promote the expression of CD163 and CD206. This leads to the activation of macrophages and their pro-tumor effect, thereby promoting epithelial-mesenchymal transition (EMT) in lung cancer and inhibiting cell apoptosis (173, 174).

PADI4 can mediate the proliferation and metastasis of cancer cells by promoting NETs. In breast cancer, PADI4 can also mediate the formation of cancer extracellular chromatin networks (CECNs) and promote lung metastasis of breast cancer (175). In PDAC, PADI4 activates pancreatic stellate cells through DNA released by neutrophils in NETs, promoting PDAC proliferation and metastasis (176).

PADI4 can participate in the proliferation and metastasis of cancer cells through certain signaling pathways. The overexpression of PADI4 increases the level of nuclear GSK3β protein, thereby inhibiting the epithelial-mesenchymal transition induced by TGF-β signaling (177). In gastric cancer, PADI4 can accelerate GC metastasis by promoting IL-8 and can also promote proliferation (178, 179). In osteosarcoma cells, PADI4 can also stimulate Wnt/β-Catenin and MEK/ERK signaling and promote proliferation (180). In nasopharyngeal carcinoma, PADI4 can activate the PI3K/AKT pathway to promote proliferation (181).

In addition, PADI4 can mediate tumor resistance. In non-small cell lung cancer (NSCLC), the overexpression of PADI4 can downregulate the expression of ETS domain protein (Elk1) and inhibit EMT, thereby reducing the drug resistance of NSCLC. In HCC cells, the overexpression of PADI4 can mediate protective autophagy, leading to resistance to chemotherapeutic drugs (182). In NPC cells, PADI4 overexpression can inhibit DNA damage. Additionally, PADI4 can downregulate the expression of p21 and activate the mTOR signaling pathway to induce radiation resistance (183–185). In breast cancer cells, PADI4 can also reverse multidrug resistance (MDR) by activating GSK3β/p53. In CRC cells, PADI can promote the migration and growth of GSK3β by promoting the nuclear transport of nuclear cyclin-dependent kinase inhibitor 1 (CDKN1A) ubiquitin-dependent proteasome degradation (186).

PADI4 can also mediate tumor-associated angiogenesis. In breast cancer and liver cancer, PADI4 can promote angiogenesis and tumor growth (187). In gastric cancer, PADI4 promotes gastric tumorigenesis and angiogenesis by upregulating CXCR2, KRT14, and TNF-α expression (53).

Therefore, PADI4 acts as an oncogene or tumor suppressor gene in tumors mainly by mediating tumorigenesis, proliferation, migration, angiogenesis and drug resistance.

In summary, the members of the PADI family (at present, there is no report on the relationship between PADI6 and tumors) could participate in tumor progression through various mechanisms. Thus, it plays an important role in tumor pathology and provides a target for tumor treatment.

## The PADI family and reproductive development-related diseases

Studies have shown that the expression of the PADI family in reproductive organs and germ cells is crucial for reproductive developments. In Sertoli cells, the specific expression of PADI2 is involved in testis development by mediating the regulation of target genes by SOX9 regulation (12). PADI6 is associated with the ovarian reserve (OR) in the primordial follicle pool (31). During early embryonic development, PADI1 transactivates the early embryonic genome by catalyzing histone tail citrullination. PADI6 ensures an adequate ribosome supply by promoting oocyte cytoplasmic lattice (CPL) formation and promoting the progression of early embryonic development (7, 188).

However, the abnormal expression of PADI family members can trigger reproductive development-related diseases. First, polymorphisms in PADI6 are associated with sexual developmental disorders (189). Second, PADI6 is one of the genes encoding the subcortical maternal complex (SCMC), which is necessary for oocyte maturation and early embryonic development. Therefore, the loss and mutation of PADI6 destabilizes SCMC, resulting in abnormal oocyte maturation, fertilization failure, early embryonic developmental arrest, multilocus imprinting disorder, molar pregnancy, miscarriage, and female infertility (32, 190–207).

In conclusion, the PADI family is an important regulator that ensures the normal progression of reproductive development.

## The PADI family and other disorders

First, the PADI family can participate in the differentiation of the epidermis and hair follicles. In the process of epidermal differentiation, PADI1 and/or PADI3 can promote keratinization of the epidermis through autophagy and promote the preservation of keratinocytes through citrullinated keratin to enhance the ability of the body to adapt to different environments (27, 208). In the differentiation of hair follicles, PADI1 promotes the ability of β-catenin to stimulate hair follicle differentiation, and PADI2 is involved in the development of hair follicles (209, 210). Moreover, PADI3 is involved in hair shaft formation, and its loss causes morphological changes in hair, leading to the development of uncombable hair syndrome (UHS) and central centrifugal cicatricial alopecia (CCCA) (211).

In addition to the above, the PADI family may also be involved in the occurrence of other types of diseases. In the heart, the citrullination of myofilament proteins by the PADI family results in cardiac contractility impairment and reduced cellular sensitivity to Ca^2+^, leading to heart failure (HF) (212). In the retina, PADI2 and PADI4 are associated with age-related macular degeneration (AMD) in the human retina through their citrullination of proteins (213, 214). In addition, PADI2 can promote the formation of elastic fibers by citrullinating Fibulin-5 (FBLN5). PADI2 can also inhibit the senescence-related secretory phenotype (SASP) by inhibiting the NFκB signaling pathway, thereby delaying the senescence of osteoblasts, increasing resistance to pneumonia and improving environmental adaptability (215–217). Moreover, PADI4 can reduce susceptibility to tuberculosis (218).

## Application of the PADI family in disease treatment

As mentioned above, the PADI family is mainly involved in inflammatory autoimmune diseases, cancer and reproductive development-related diseases and plays an important role in the occurrence of these diseases. Therefore, in recent years, an increasing number of researchers have explored the possibility of the PADI family as a target in the develop of drugs, as shown in **Table 3**.

**Table 3:** Application of the PADI family in disease treatment.

Disease type	Mechanism	Drug
Acute myeloid leukemia cells	Induces ER stress by targeting PADI2	BB-Cl-Amidine (219)
CRC resistant cells	Reverses MDR by targeting PADI2 and PADI4	NTZ (220)
Acute lung injury	Regulates the localization of p65 in the nucleus of epithelial cells by targeting PADI4	TDFA (221)
Multiple sclerosis	Targets PADI2	Compound 23 (222)
Tamoxifen-resistant breast cancer cells	Reduces tamoxifen resistance by targeting PADI2	Cl-amidine (165)
Remote Lung Injury	Reduces NETs by suppressing PADI4	GSK484 (128)
Atherosclerosis	Inhibits VCAM-1 expression and adhesion of monocyte to vascular smooth muscle cells through MAPK and PADI4-dependent NF-kB and AP-1 pathways	Ramalin (223)
Breast cancer	Influences the expression of tumor-related cell cycle genes (p21, GADD45α and Ki67)	Cl-amidine (148)
MM	Blocks the formation of NETs by suppressing PADI4	BMS-P5 (224)

## Discussion

**Figure 5:**
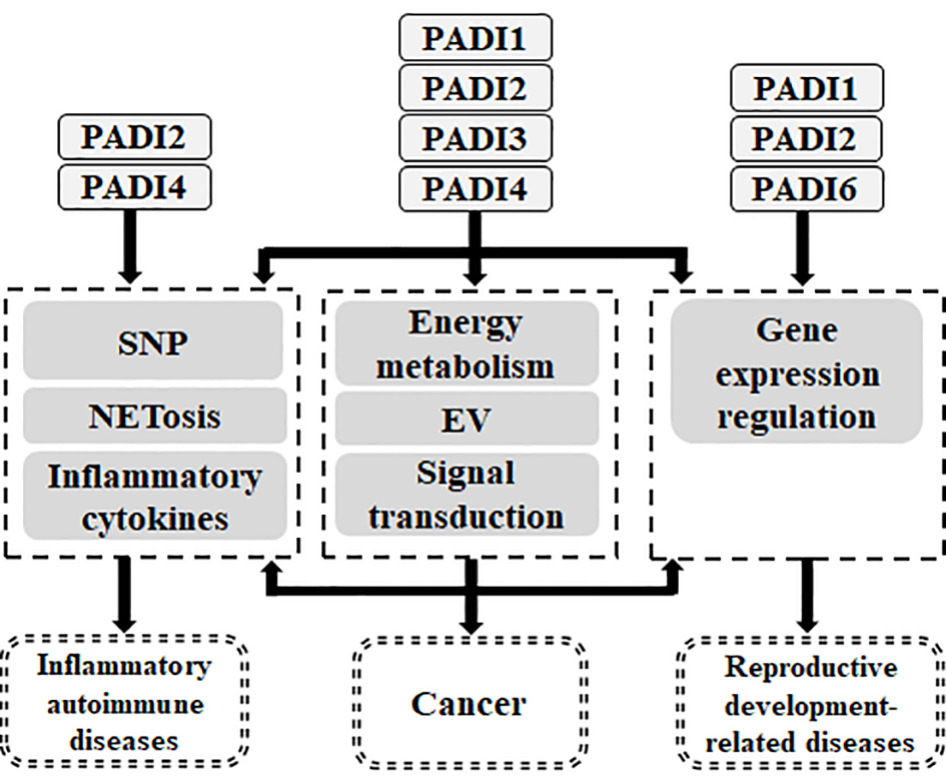
Relationship between PADI family and human diseases. The genetic variation of PADI family will endow with the susceptibility of inflammatory autoimmune diseases and cancer. PADI family can participate in inflammatory autoimmune diseases through NETosis and inflammatory cytokines. PADI family can participate in cancer through NETosis, inflammatory cytokines, energy metabolism, EV, signal transduction and gene expression regulation. PADI family can participate in reproductive and development-related diseases through gene expression regulation.

Studies have shown that the calcium ion dependent enzyme PADI family targets protein substrates through its citrullination. First, there is crosstalk between PADI family members and methylation by targeting histones, and thus they play a role as epigenetic modifying enzymes. Secondly, the PADI family can regulate gene expression and signal transduction by targeting non-histone proteins. Therefore, the PADI family can participate in various physiological processes through the citrullination of proteins, which is associated with the occurrence of diseases (**Figure 5**).

In arthritis, the genetic variation of PADI2 and PADI4 can confer RA susceptibility, and they can aggravate arthritis by promoting the secretion of NETosis and inflammatory cytokines. In neurodegenerative diseases, the high expression of PADI2 leads to the abnormal accumulation of citrullinated proteins and promotes their malignant progression, while PADI4 can lead to inflammation through citrullinated histone H3, thus promoting the progression of neurodegenerative diseases. However, PADI2, PADI3 and PADI4 can also inhibit the progression of neurodegenerative diseases by participating in the differentiation, apoptosis and senescence of nerve cells. In atherosclerosis and thrombosis, PADI4 can lead to an inflammatory response and thrombosis by promoting NETosis. In SLE, PADI2 and PADI4 can promote its progression through the immune response. In addition, the genetic variation of PADI4 can confer SLE susceptibility and promote its deterioration by promoting NETosis. In systemic infection, PADI2 and PADI4 can lead to the deterioration of infection by promoting NETosis, and PADI2 can also lead to the deterioration of infection by inducing cell apoptosis. Therefore, in inflammatory autoimmune diseases, the PADI family mainly participates in disease progression through PADI2 and PADI4.

In cancer, PADI1 can promote tumor proliferation, migration, invasion and drug resistance by promoting energy metabolism and signal transduction (MEK1-ERK1/2-MMP2, ERK1/2-p38 and PADI1-MAPK-MMP2/9), thus it acts as oncogene in cancer. PADI2 can regulate gene expression, energy metabolism, inflammatory response and signal transduction (MEK1/ERK1/2/IGF2BP1/SOX2, JAK2/STAT3, Wnt/β-Catenin, EGF, Dll4/Notch1) to mediate the proliferation, migration, invasion and drug resistance of tumor cells, thus acting as an oncogene and tumor suppressor gene. PADI3 can affect the proliferation and invasion of tumor cells by regulating energy metabolism, EV and signal transduction (Sirt2/p21/AKT/Snail, Hsp90/CKS1), thereby inhibiting or promoting cancer. PADI4 can affect tumorigenesis by regulating cell activity, cell cycle progression and differentiation. PADI4 can also regulate gene expression, NETs and signal transduction (GSK3β/TG-β、Wnt/β-Catenin、MEK/ERK、PI3K/AKT、GSK3β/P53) to affect the proliferation, migration, invasion, drug resistance and angiogenesis of tumor cells. Therefore, PADI4 can promote and inhibit cancer. As mentioned earlier, the role of the PADI family (except PADI6) in cancer is still under debate. PADI family members can function as oncogenes and as tumor suppressors.

Among the diseases related to reproductive development, PADI1, PADI2 and PADI6 are necessary to ensure normal reproductive development. However, current research shows that the abnormal expression of PADI6 can lead to abnormal oocyte maturation, fertilization failure, early embryo development stagnation, multiple site imprinting disorder, hydatidiform mole, abortion and female infertility, and the genetic variation of PADI6 is related to sexual development disorder.

In summary, PADI family members can play different roles in different disease types. On one hand, they can ensure the normal progression of various physiological activities in the body and prevent the occurrence of diseases. On the other hand, the abnormal expression of PADI family members can lead to disorders in physiological processes in the body and promote the occurrence of diseases. However, there is no doubt that due to the unique function and physiological roles of PADI family members, their potential as therapeutic targets in disease will receive increasing attention.

As mentioned above, PADI2 and PADI4 are the most studied members of the PADI family. They are mainly involved in the development of inflammatory autoimmune diseases and cancer, and they can be used as targets of disease treatment for drug development (**Table 3**).

At present, the mechanism by which the PADI family is involved in human physiological processes and diseases is still being investigated. Among its members, PADI2 and PADI4 are the most studied. They are mainly involved in the development of inflammatory autoimmune diseases and cancer, and they are used as targets of disease treatment for drug development (**Table 3**). In recent years, the important role of the PADI family in diseases has attracted people’s attention. In the future, clarifying the functions of PADI family members and the molecular mechanism of their role in various diseases will be the focus of research.

In this study, we systematically describe the function of PADI family members and their specific mechanism and research progress in various diseases, hoping to provide a reference for the study of the PADI family and promote the application of PADI family members as therapeutic targets for clinical diseases. The names of all proteins involved in this article are shown in **Table 4**.

**Table 4:** Genes involved in this study.

PADI1	PADI2	PADI3	PADI4	PADI5
NFKB1	MZF1	Sp1/Sp3	P2RX7	SOX9
FOXL2	NFYA/NFYB/NFYC	p53	JUN/FOS	NOBOX
AR	ER	p21	DNMT3A	LMNA/LMNB1
HMGB1	NLRP3	VIM	GATA3	TNF
CCL2	BRD4	PAI2	IL6ST	PKM
TLR4	SYVN1	FN1	HLA-DRB1	GFAP
TLR7	IKBKG	CAMP	GADD45α	MKI67
HMGA1	MYCN	IGF1	EPO	ACSL4
BIRC3	CA9	CXCR2	MSN	SIRT2
HSP90AA1/HSP90AB1	CKS1B	NANOG	POU5F1	ELK1
KRT14	SOX9			

## Conclusion

In this review, firstly we discussed the difference between five PADI family members, which showed that they have different localization, expression patterns and functional specificity. Secondly, we discussed the genetic variation of PADI family members (PADI2 and PADI4) is related to disease susceptibility. In addition, the PADI family can participate in the regulation of gene expression, the formation of neutrophil extracellular traps, the secretion of inflammatory cytokines, energy metabolism and the release of extracellular vesicles and other important physiological processes, so that it can participate in disease progression (including inflammatory autoimmune diseases, cancer, and reproductive development-related diseases).

## Author contributions

CZ, ZC and CL prepared the Manuscript, Tables and Figures, CL and ZC provided fundings. All authors read and approved the final manuscript.

## Glossary

**Table:**

PADI	peptidyl arginine deiminase
OR	ovarian reserve
SNP	single nucleotide polymorphism
NETs	neutrophil extracellular traps
AR	androgen receptor
ER	estrogen receptor;
H3R26Cit	Histone H3 26 arginine citrullinated
RNAP2-CTD	RNA polymerase II C-terminal domain;
DNMT3A	DNA methyltransferase 3A
NETosis	the process of neutrophil extracellular traps formation;
CitH3	citrullinated histone H3
HMGB1	high mobility group box 1
NLRP3	nod-like receptor protein 3;
GATA3	GATA Binding Protein-3
RORgt	retinoic acid-related orphan receptor gamma t
IL	interleukin;
NF-kB	nuclear factor-kappa B
TNF-a	tumor necrosis factor-a
CCL2	C-C motif chemokine ligand 2
PAI-2	Plasminogen activator inhibitor-2
PSMB1	Proteasome Subunit Beta
Tal1	T-cell acute lymphocytic leukemia 1
BRD4	bromodomain-containing protein 4
E2F-1	E2F transcription Factor 1
CCL3	C-C motif chemokine ligand 3
PKM2	citrulline pyruvate kinase M2
TINCR	tissue differentiation-inducing nonprotein coding RNA
ACLY	ATP Citrate Lyase
MAPK	mitogen activated protein kinase
MMP2/9	matrix metalloproteinase-2/9
EV	Extracellular vesicle
RA	rheumatoid arthritis
FLS	fibroblast-like synovial cells
SF	synovial fluid
ACPAs	anti-citrullinated protein antibodies
TLR	Toll-like receptor
MIP1b	macrophage inflammatory protein Ib
IFNa	interferon a
METs	monocyte extracellular traps
RASF	rheumatoid arthritis synovial fibroblasts
SYVN1	synoviolin 1
FN	fibronectin
ADAMTS4	thrombospondin motifs 4 human leukocyte antigen-DRB1
RA-ILD	rheumatoid arthritis-related interstitial lung disease
OA	osteoarthritis
JIA	juvenile idiopathic arthritis
GIA	glucose 6-phosphate isomerase-induced arthritis
AS	ankylosing spondylitis
hMSCs	human mesenchymal stem cells
PTPN22	Protein Tyrosine Phosphatase Nonreceptor 22
AD	Alzheimer’s disease
PD	Parkinson’s disease
ALS	amyotrophic lateral sclerosis
GFAP	glial fibrillary acidic protein
CNS	central nervous system
MS	multiple sclerosis
PTLD	posttreatment Lyme disease
XDP	X-linked dystonia Parkinson’s disease
PFC	prefrontal cortex
AIF	apoptosis-inducing factor
VOC	vaso-occlusive crisis
SCD	sickle cell disease
HIT	heparin-induced thrombocytopenia
ANCA-AAV	anti-neutrophil cytoplasmic antibodyassociated vasculitis
SLE	systemic lupus erythematosus
JLP	JNK-related leucine zipper protein
TNFAIP3	TNF alpha induced protein 3
LN	lupus nephritis
IR	ischemia-reperfusion
NEMO	nuclear factor kappa B essential modulator
HS	hidradenitis suppurativa
RSV	respiratory syncytial virus
MG	meibomian gland
MGD	meibomian glands dysfunction
HSCs	hepatic stellate cells
HRV	human rhinovirus
cFBG	Citrullinated fibrinogen
MEK1	mitogenactivated protein kinase 1
ERK1/2	extracellular signal-regulated kinase 1/2;
PAAD	pancreatic ductal adenocarcinoma
NPC	nasopharyngeal carcinoma
PC/PDAC	pancreatic cancer/pancreatic ductal adenocarcinoma
LSCC	laryngeal squamous cell carcinoma
P-TEFb	positive transcriptional elongation factor b;
HMGA1	high mobility group A1
IGF-1	Insulin-Like Growth Factor -1
ACSL4	Acyl-CoA Synthetase Long-Chain Family Member 4
BIRC3	Baculoviral IAP Repeat containing 3
CA9	carbonic anhydrase 9
CXCR2	CXC chemokine receptor 2
BMMSCs	bone marrow mesenchymal stem cells
IGF2BP1	insulinlike growth factor-II binding protein 1
EGF	epidermal growth factor
Akt	protein kinase B/PKB
HSP90	Heat shock protein 90
CKS1	cyclin kinase subunit 1
OSCC	oral squamous cell carcinoma
ESCC	esophageal squamous cell carcinoma
Bmi-1	B-cell-specific Moloney leukemia virus insertion site 1
IRF-5	interferon regulatory factor 5
EMT	epithelial-mesenchymal transition;
CECNs	cancer extracellular chromatin networks
GSK3b	glycogen synthase kinase 3 beta
TGF-b	transforming growth factor-b
GC	gastric cancer
PI3K	phosphoinositide 3-kinase
NSCLC	non-small cell lung cancer
Elk1	ETS domain protein
HCC	hepatocellular carcinoma
MDR	multidrug resistance;
CDKN1A	cyclin-dependent kinase inhibitor 1
CRC	CRC/colon cancer
MM	multiple myeloma
Dll4/Notch1	Delta-like ligand 4/Notch homologue 1
EPO	erythropoietin
SOX9	SRY-related high mobility group box gene 9
CPL	oocyte cytoplasmic lattice
SCMC	subcortical maternal complex
UHS	uncombable hair syndrome
CCCA	central centrifugal cicatricial alopecia
HF	heart failure
AMD	age-related macular degeneration.
